# *Drosophila* Mutant Model of Parkinson's Disease Revealed an Unexpected Olfactory Performance: Morphofunctional Evidences

**DOI:** 10.1155/2016/3508073

**Published:** 2016-08-28

**Authors:** Francescaelena De Rose, Valentina Corda, Paolo Solari, Patrizia Sacchetti, Antonio Belcari, Simone Poddighe, Sanjay Kasture, Paolo Solla, Francesco Marrosu, Anna Liscia

**Affiliations:** ^1^Department of Biomedical Sciences, University of Cagliari, Cagliari, Italy; ^2^Department of Life and Environmental Sciences, University of Cagliari, Cagliari, Italy; ^3^Department of Agricultural Biotechnology, Section of Plant Protection, University of Florence, Firenze, Italy; ^4^Pinnacle Biomedical Research Institute, Bhopal, India; ^5^Department of Public Health, Clinical and Molecular Medicine, University of Cagliari, Cagliari, Italy

## Abstract

Parkinson's disease (PD) is one of the most common neurodegenerative diseases characterized by the clinical triad: tremor, akinesia, and rigidity. Several studies have suggested that PD patients show disturbances in olfaction as one of the earliest, nonspecific nonmotor symptoms of disease onset. We sought to use the fruit fly* Drosophila melanogaster* as a model organism to explore olfactory function in LRRK* loss-of-function* mutants, which was previously demonstrated to be a useful model for PD. Surprisingly, our results showed that the LRRK mutant, compared to the wild flies, presents a dramatic increase in the amplitude of the electroantennogram responses and this is coupled with a higher number of olfactory sensilla. In spite of the above reported results, the behavioural response to olfactory stimuli in mutant flies is impaired compared to that obtained in wild type flies. Thus, behaviour modifications and morphofunctional changes in the olfaction of LRRK* loss-of-function* mutants might be used as an index to explore the progression of parkinsonism in this specific model, also with the aim of studying and developing new treatments.

## 1. Introduction

The olfactory system represents the most common and ancient sensory system within the animal kingdom, from single-celled organisms through higher animals [1], and one of the most important sensory modalities, due to its crucial role in conveying information about the external world to the nervous system. In this respect, dysfunctions of the olfactory system may have negative effects on the quality of life [2] and in some cases it has been associated with a higher mortality risk [3]. Several studies have demonstrated the connection between smell dysfunctions and Parkinson's disease (PD) [4–9]. Although PD is usually considered as a movement disorder, several studies have shown that PD nonmotor symptoms may have greater impact on quality-of-life measures, institutionalisation rates, or health economics (for a review, see [10]). Moreover, among PD nonmotor disorders, olfactory disturbances may often precede motor symptoms, representing a potential predicting marker for PD [11]. Indeed, the same presence of Lewy bodies in the substantia nigra [12], strictly related to the onset of typical PD motor symptoms, is a later step in the progression of the pathology. Indeed, initial presence of Lewy bodies has been clearly observed in the medulla oblongata and in the olfactory bulb, thus preceding the successive involvement of midbrain, diencephalic nuclei, and neocortex [13].

Although it is now clear that PD is associated with a combination of risk factors, including environmental noxious agents and genetic predisposition [14], which of the many risk factors may be associated with the timing of disease onset is not known.

Furthermore, regarding genetic predisposition, only a limited percentage is due to monogenic forms of the disease [15]. Among the several genetic mutations implicated in PD aetiology, those associated with the leucine-rich repeat kinase 2 gene (LRRK2) are actually known as responsible for the most common familial and sporadic disorder cases [16–18]. Two more common variations in the* LRRK2* gene have been described: G2019S and G2385R [19]. Among them, the most common (G2019S) accounts for 3–6% of familial dominant PD and for 1-2% of sporadic forms with a north-south gradient of G2019S frequency in European countries and reaching frequency up to 41% in North African cases [20], while the second variation (G2385R) is common mainly in Asian populations [19, 21]. To date, apparent discrepancies between these variations have been reported, suggesting a more complicated phenotype in the G2385R mutation carriers than in* LRRK2* G2019S mutation carriers [19].

Although the evidence about LRRK2 role in inflammatory processes and in the endolysosomal system and cytoskeleton impairment has been identified [22], its involvement in PD development is still not fully defined. Interestingly, different studies highlighted that carriers of a given LRRK2 mutation show a clinical and pathological variability in the manifestation of the disorder, with nigral degeneration associated with absence and limited or wide diffusion of Lewy bodies [16, 17, 23–25]. This phenotypic and pathological variability, however, is found in nearly all monogenic causes of PD, even within nuclear families (e.g., SNCA A53T) [26].

Some variability has also been observed regarding nonmotor symptoms, where LRRK2 G2019S olfactory phenotype may be less pronounced than idiopathic PD and possibly G2385R mutation, and it is not clear that all G2019S mutation carriers have olfactory impairment [19, 27–36].

Given the above reported phenotypic variability in PD olfactory dysfunction, it seems of particular interest to have a simple model to study this nonmotor symptom, particularly in a LRRK2 mutant.

The LRRK2 gene coding for unusually large protein composed of 2527 amino acids is widely expressed in the brain and other organs [37–40]. The LRRK2 roles, listed in the Berwick and Harvey review [22], include neurogenesis and neurite outgrowth, cytoskeleton assembly, endocytosis/vesicles trafficking, and autophagy coordination.

LRRK2 is a member of the ROCO protein family characterized by the presence of two conserved domains: a Roc (Ras in complex proteins) domain belonging to the Ras/GTPase superfamily and a COR domain (C-terminal of Roc). Three further conserved domains have been described: a leucine-rich repeat (LRR); a tyrosine kinase catalytic domain (MAPKKK); and finally a WD40 domain [17]. This latter is known to be crucial in several basic cell functions such as vesicle sorting during endocytosis and exocytosis of synaptic vesicles as well as vesicle-mediated transport and cytoskeleton assembly [41, 42].

Recently, De Rose et al. [43] reported that* Drosophila* LRRK2* loss-of-function* mutant for deletion of the domain WD40 (LRRK2^WD40^; LRRK^ex1^ mutant, [44, 45]) showed a motor age-dependent impairment and a correlated mitochondrial impairment in the thoracic ganglia. Besides, a mitochondrial impairment was detected in the antennal lobe area, where olfactory signal from the peripheral chemoreceptors projects. Similarly, a morphological impairment was reported by Poddighe et al. [46] in another* Drosophila* mutant such as PINK1^B9^ which also showed a decrease in the olfactory detection, both in the electrophysiologically recorded olfactory signals and in the olfactory behaviour.

On this basis, the aim of this study was to estimate any possible olfactory dysfunction in the* Drosophila* LRRK2 model of PD correlated to LRRK2 mutation (LRRK^WD40^). This model most closely approximates human LRRK2 G2385R mutations, which occur in the human WD40 domain and are a risk factor for PD. The results showed that the LRRK^WD40^ mutant presents an unexpected dramatic increase in the amplitude of electrophysiological responses, but a decrease in the olfactory discrimination, with respect to wild type flies (WT).

## 2. Materials and Methods

### 2.1. Insects

For these experiments adult wild type (WT; Canton-S) and LRRK^WD40^ mutant (LRRK^ex1^, #34750, from Bloomington Stock Center)* Drosophila melanogaster* (Dm) males were used. Soon after emergence from pupae, WT or LRRK^WD40^ mutant males were separated from females. WT and mutant flies were reared on a standard cornmeal-yeast-agar medium in controlled environmental conditions (24-25°C; 60% relative humidity; light/dark = 12/12 hours). Flies ranging 10–15 days in age were tested according to previous experiments [43].

### 2.2. Electroantennograms (EAGs) Recordings

Electroantennogram (EAG) recordings were performed* in vivo* as previously described [47, 48].

By taking into account the circadian cycle in olfactory sensitivity [49], WT and mutant flies were always tested in parallel. Briefly, living 10- to 15-day-old male flies were singly inserted in a 100 *μ*L truncated plastic pipette, with the head protruding at the tip. The preparation was fixed with dental wax on a microscope slide and positioned under the viewer of an Olympus BX51WI light microscope (Olympus, Tokyo, Japan). Glass capillaries with a silver wire were filled with a conductive 0.15 M NaCl solution. The recording glass electrode was gently placed on the tip of the antennal funiculus, whereas the reference electrode was inserted in the compound eye. The EAG signal was amplified with an AC/DC probe and then acquired with an IDAC-4 interface board (Syntech, Hilversum NL). A charcoal purified and humidified airflow was constantly blown over the antennae (speed 0.5 m/s) via a glass tube, placed approximately 1 cm from the antenna. The tip of a Pasteur pipette containing an odour-loaded filter paper (5 mm × 25 mm) was inserted into a small hole in the glass tube. Odour stimulation was administered by injecting a puff of purified air (0.5 s at 10 mL/s airflow) through the pipette using the stimulus delivery controller (Syntech).

Odour stimuli tested were dissolved in hexane and presented in series from minor to higher concentration (resp., 0.01, 0.1, 1, and 10% v/v). 1-Hexanol was chosen for its well-known stimulant activity in* Drosophila* [50–52], and 1-linalool, a terp commonly found in plants, was chosen for its capability to excite olfactory sensory neurons in different species of insects [53]. As the standard reference, 1-hexanol was administered at the 10% v/v dilution at the beginning of the experiments, to confirm the activity of the antenna. Both 1-hexanol and 1-linalool were purchased from Sigma-Aldrich (Milan, Italy).

Mean values of EAG amplitude were calculated and then analysed by comparing the results obtained in LRRK^WD40^ mutant groups with the age-matched WT control group.

The significance of differences was tested by repeated-measures ANOVA or one-way ANOVA (followed by HSD* post hoc* test) with a threshold level of statistical significance set at *P* ≤ 0.05 (statistical software package). EAG results are expressed as average values + SEM and represented by histograms.

### 2.3. Olfactory Behaviour

Free-walking bioassays were performed following the experimental procedures used by Dekker et al. [54]. Briefly, males of WT and LRRK^WD40^ were given the opportunity to choose between vials containing water with or without odour (1-hexanol or 1-linalool). Two 4 mL glass vials were placed symmetrically and equally spaced in a large Petri dish (the arena) and then fitted with truncated pipette tips. The vials were filled with 300 *μ*L of water with 0.25% Triton X with or without the odorant. On the basis of the EAG response, the odour dilution chosen to trap the flies was 0.1% for both compounds. The dehydration of flies was prevented by placing a cotton ball with 3 mL of water in the arenas. Flies were starved for 9 hours prior to starting the experiments. Twelve bioassays were carried out for each experimental group of flies (15 flies per arena) and for each odour source and then replicated three times. Bioassays were performed in controlled environmental conditions and lasted 18 hours [54] that comprised the most active phase of olfactory sensitivity [49]. The attraction index (AI) was calculated as follows: (*T* − *C*)/(*T* + *C* + *NR* − *D*), in which *T* is the number of flies in the treatment, *C* is the number in the control, *NR* is the number of living flies remaining in the arena, and *D* is the number of dead flies.

Data obtained were expressed as average of percentages of flies reaching the treatment vial (with 1-hexanol or 1-linalool) or the control vial (water) and statistically evaluated by means of Student's *t*-test (Statistical software package) with a 95% confidence level.

### 2.4. Morphological Observations

For scanning electron microscopy (SEM), 4–6-day-old flies were anesthetized with carbon dioxide, immediately immersed in hexane, shaken for 3 minutes to remove the external wax layer, then dehydrated in a graded ethanol series for ten minutes each concentration up to absolute ethanol, subsequently air-dried, and finally glued to stubs and gold coated. At least ten specimens were prepared and observed using a FEI Quanta 200 high-vacuum SEM at the Department of Tree Science, Entomology and Plant Pathology “G. Scaramuzzi,” University of Pisa. The density of antennal sensilla in both WT and LRRK^WD40^ mutant males was determined by counting the number of different types of sensilla present in a sample area enclosed by an electronic square frame (1,000 *μ*m^2^) applied to the SEM screen in the central part of the flagellum. This area of the flagellum was chosen since it was plain displayed in all samples and the arrangement of the antennal structures allowed to count easily the number of each type of sensilla included by the frame. Moreover, in this area all kinds of sensilla were present, with the exception of one type (large basiconic sensilla) located mainly in the basal part of the flagellum [53]. Sensilla were counted in the flagellar area of one antenna per specimen; differences in the number of the three types of sensilla counted in the analysed area of males of the two strains were evaluated by Student's* t-*test with a 95% confidence level (Statistica 6.0, StatSoft, Italy). Higher magnification was used to study in detail the morphology of different types of sensilla.

## 3. Results

### 3.1. LRRK^WD40^ Mutants Show Enhancement of the EAG Response to Both 1-Hexanol and 1-Linalool

**Figure 1:**
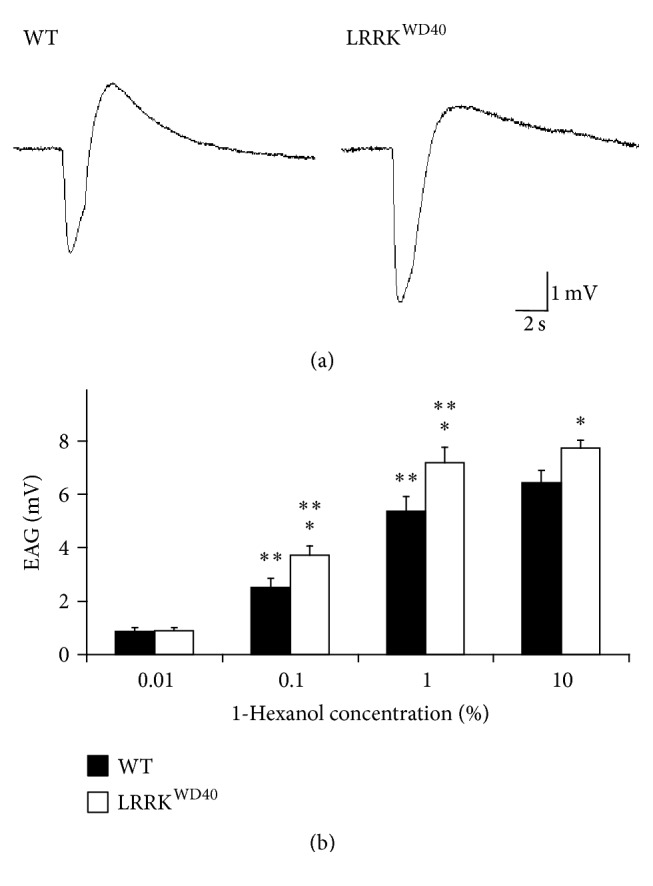
Electroantennogram (EAG) responses to 1-hexanol. Sample EAG recordings (a) and EAG amplitude values (b) elicited by stimulation with the different concentrations of 1-hexanol (0.01, 0.1, 1, and 10% v/v) in male antennae of WT and LRRK^WD40^ mutant flies. Mean values + SEM from 24–26 antennae for each stimulus concentration and insect sample. *∗* and *∗∗* indicate significant differences (*P* < 0.05; HSD* post hoc* test subsequent to one-way ANOVA or repeated-measures ANOVA) from WT and from preceding concentration of same stimulus, respectively.

**Figure 2:**
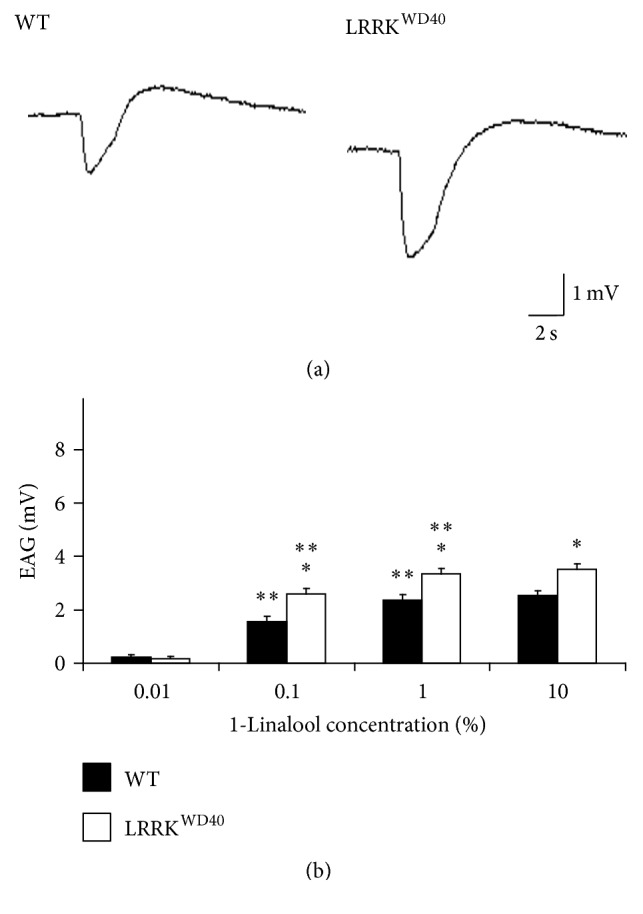
Electroantennogram (EAG) responses to 1-linalool. Sample EAG recordings (a) and EAG amplitude values (b) elicited by stimulation with the different concentrations of 1-linalool (0.01, 0.1, 1, and 10% v/v) in male antennae of WT and LRRK^WD40^ mutant flies. Mean values + SEM from 24–26 antennae for each stimulus concentration and insect sample. *∗* and *∗∗* indicate significant differences (*P* < 0.05; HSD* post hoc* test subsequent to one-way ANOVA or repeated-measures ANOVA) from WT and from preceding concentration of same stimulus, respectively.

As shown in Figures 1(a) and 2(a), the olfactory stimulations of flies' antennae with both 1-hexanol and 1-linalool (0.01, 0.1, 1, and 10% v/v) consistently elicited responses with the typical EAG waveform, that is, a rapid depolarization followed by a slower recovery phase, ending with the hyperpolarized wave before complete reversal to the baseline, in both WT and in LRRK^WD40^ mutants, the repolarization phase in LRRK^WD40^ being slower than WT.

Contrary to expectations, the EAG response values elicited after stimulation with both 1-hexanol (Figure 1) and 1-linalool (Figure 2) in LRRK^WD40^ mutants were higher than those obtained in WT specimens, exhibiting a dose response for both stimuli administered, although this tendency was more evident for 1-hexanol. In detail, LRRK^WD40^ flies showed a significant increase (*P* < 0.05) in the EAG amplitudes with respect to WT after stimulation with 1-hexanol and 1-linalool for all concentrations tested, except the lowest (Figures 1 and 2).

### 3.2. LRRK^WD40^ Mutants Show Impairment of the Behavioural Response to Both 1-Hexanol and 1-Linalool

**Figure 3:**
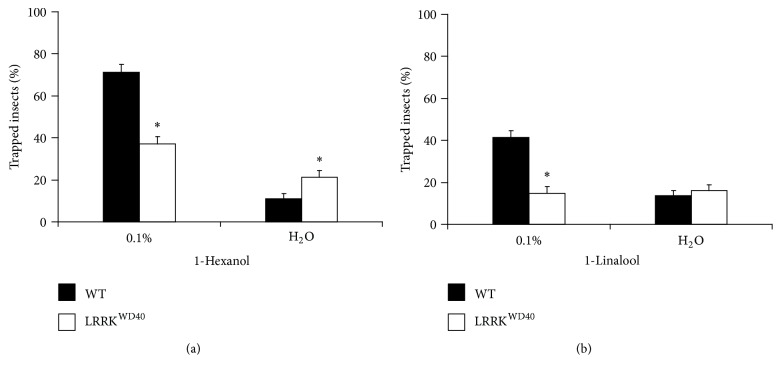
Behavioural olfactory response to 0.1% v/v 1-hexanol (a) and 0.1% v/v 1-linalool (b) in WT and LRRK^WD40^ mutant flies. Mean values of trapped males + SEM; experiments in triplicate; *n* = 12 bioassays for each experimental group of flies, *n* = 15 flies per arena. *∗* indicates significant differences (*P* < 0.05; Student's* t*-test) with respect to WT.

As for the EAG tests, free-walking bioassays were performed on WT and LRRK^WD40^ adult males, by testing the responses to 1-hexanol or 1-linalool, both at the dilution 0.1% v/v. Contrary to electrophysiological results, LRRK^WD40^ males presented a behavioural impairment, with a significant decrease in the behavioural scores for both odours with respect to the WT control groups (Figure 3). In fact, in the trap assays with 1-hexanol, the percentage of odour-trapped insects was 37 ± 3.7% for LRRK^WD40^ compared to 71.4 ± 3.7% for WT control groups; in the case of 1-linalool, only 14.8 ± 3.3% of LRRK^WD40^ mutants were attracted in the vial with the odour as compared to 41.4 ± 3.2% of WT control groups.

As for the water response, the numbers of trapped flies in the blank baits were higher for LRRK^WD40^ compared to WT (21.3 ± 3 and 11 ± 2.3%, resp., *P* < 0.05) in response to 1-hexanol, while the number of insects trapped in the blank did not differ in response to 1-linalool. Moreover, in the latter, no difference exists between mutant insects trapped by the odour with respect to the blank.

### 3.3. Morphological Observations

The unexpected increase in EAG response combined with the impairment of the behavioural response of mutants with respect to WT led us to investigate any possible variation in the antennal olfactory apparatus.

**Figure 4:**
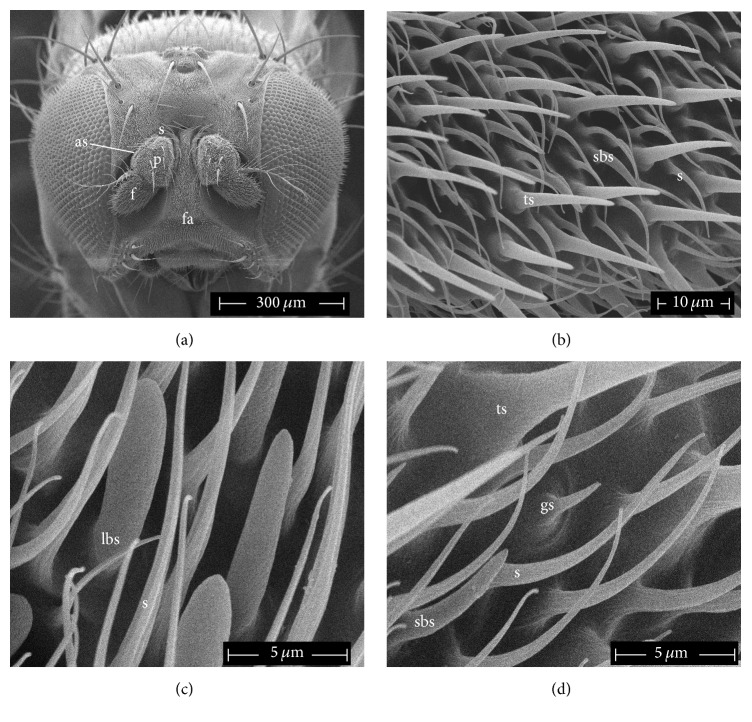
Scanning electron micrographs of* D. melanogaster* WT male. (a) Head showing the antennal pattern: scape (s), pedicel (p), antennal seam (as), flagellum (f), and face (fa). (b) Magnification of the central part of flagellum with sensilla: trichoid sensillum (ts), small basiconic sensillum (sbs), and setae (s). (c) High magnification of the basal part of the flagellum showing some large basiconic sensilla with porous wall (lbs). (d) High magnification of different types of sensilla in the central part of flagellum with a grooved sensillum (gs).

According to our results, no differences are found between WT and LRRK^WD40^ flies in the gross structure. As shown in Figure 4(a), antennae are located between the compound eyes in two pits separated by a prominent face. The antennae consist of three segments: scape (basal segment), pedicel, and flagellum. The pedicel is marked dorsally by a longitudinal antennal seam. The flagellum, the most relevant sensory area, bears different types of sensilla interspersed with cuticular setae which are in general markedly curved and furrowed. There are 4 types of sensilla: trichoid, large basiconic, small basiconic, and grooved sensilla (Figures 4(b), 4(c), and 4(d)). Trichoid sensilla are the longest, most predominant sensilla which are spread in the whole flagellum; large basiconic sensilla are present mainly in the basal part of flagellum and show a multiporous surface (Figure 4(c)), while small basiconic sensilla are present mainly in the middle and in the distal part of the flagellum. Grooved sensilla are present only in the central and in the distal part of the flagellum; they appear very small and are formed by a series of 6–8 finger-like projections (Figure 4(d)).

However, as shown in Table 1, significant differences were found in the mean number of different types of sensilla counted in the selected area of flagellum between WT and LRRK^WD40^ mutants.

**Figure 5:**
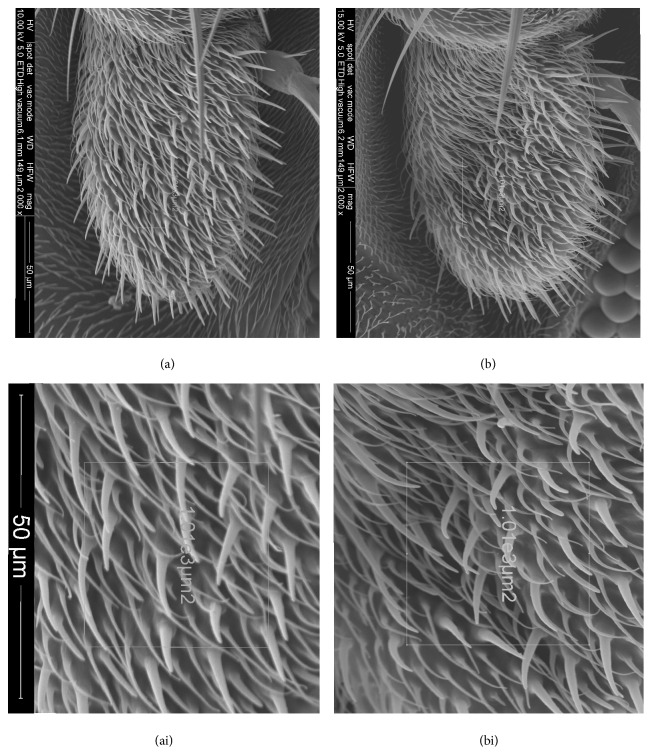
Scanning electron micrographs of flagellum of* D. melanogaster* WT (a) and LRRK^WD40^ mutant (b); (ai and bi) the respective magnifications of the central area with the frame used to count the density of sensilla.

In detail, as a general pattern, mutants show a higher number of all sensilla in the considered area than WT flies, although these differences are evident only at high magnification (Figure 5). As a matter of fact, the number of trichoid sensilla in LRRK^WD40^ mutants males exceeded the WT number with highly significant difference (*P* ≤ 0.001). Similarly, also small basiconic and grooved sensilla were present in LRRK^WD40^ mutants males with a slightly higher density than in the WT males (*P* ≤ 0.05).

## 4. Discussion

The aim of this study was to estimate in the PD translational model* Drosophila melanogaster* any possible olfactory dysfunction correlated to a specific LRRK2 mutation (LRRK^WD40^) [43], by analogy with a previous study in a PINK1* Drosophila* model [9, 46].

The used LRRK2 translational model most closely approximates human LRRK2 G2385R mutation, which occurs in the human WD40 domain and is a risk factor for PD, mainly in Asian populations [21].

Contrary to PINK1^B9^ [9, 46], where the EAG response was lower with respect to the WT, our results showed that the LRRK^WD40^ mutant, compared to the WT, presents an unexpected dramatic increase in the amplitude of the EAG responses and this is coupled with a higher number of olfactory sensilla.

Nevertheless, similarly to what was observed for PINK1^B9^ [46] also the LRRK^WD40^ mutants present an impairment in the behavioural response to odours.

### 4.1. LRRK^WD40^ Mutants Show Enhancement of the EAG Response to Both 1-Hexanol and 1-Linalool

In detail, even if LRRK^WD40^ shows an increase in EAG response at any concentration to both tested stimuli (1-hexanol and 1-linalool), at the lowest one, no differences in threshold were found between mutants and WT. Despite the great variability in the sensilla number in different strains [55], we cannot exclude the notion that the electrophysiological response is due to the higher sensillar density found in the flagellum of LRRK^WD40^ compared to WT, mostly in consideration of the relevant number of trichoid sensilla which can cause an increased summed antennal sensitivity. Further, we cannot exclude a role of the perireceptor environment in the chemoreceptor response. In this respect, it is possible that in mutants more odorant binding proteins could be present and/or interact with the odorants more tightly, but the presence of a diminished concentration of the odour degrading enzymes is also possible [56–58]. Accordingly, Corvol et al. [59] found in PD patients a persistent increase in an olfactory type G-protein (G_olf_) that was first identified in the olfactory epithelium [60, 61] homologous to the olfactory sensilla in* Drosophila* antennae.

More recently, Yun and Park [62], analysing shape and amplitude of the EAG curve, indicated some parameters for the analysis of the EAG curve, with the peak amplitude being the most important one and therefore it is widely used for studying the odour detection. According to these authors, other parameters like the shape cannot be used for odorant concentration measurements but they can help in discriminating between different odorants. In this respect, our results show that EAGs evoked by 1-linalool are longer than those evoked by 1-hexanol. The overall lower amplitude of the EAG in response to 1-linalool with respect to 1-hexanol is also in accordance with the data in literature, according to which the former interacts with a reduced number of ORs with respect to the latter [50].

### 4.2. LRRK^WD40^ Mutants Show Impairment of the Behavioural Response to Both 1-Hexanol and 1-Linalool

Looking at the behavioural response to the odours, 1-hexanol attracts a number of insects statistically higher than 1-linalool, in both WT and mutant flies, but the number of LRRK^WD40^ mutants trapped is lower than WT regardless of the stimulus tested. As for the functional significance of the two stimulants, 1-hexanol was reported to involve both the appetitive and the aversive response in flies, even if with great variability in its effects [51, 63], and it interacts with a number of olfactory receptor neurons (ORNs) distributed on the small and the large basiconic and on the trichoid sensilla [50, 53, 64]. In this respect, our behavioural data point to a prevailing appetitive response to 1-hexanol, thus making the differences with the above reported data in literature. 1-Hexanol is reported to also interact with a number of olfactory receptors (ORs) located in the trichoid ones responding to pheromones [64] that could give reason for the attractive effects we observed. In other words, we suggest possible sex-related differences, that is, male used in the present study versus male and female in a 1 : 1 ratio in the study by Knaden et al. [51]. We cannot exclude other possible methodological differences among different studies. Analogous considerations can be made for 1-linalool that was previously reported to be an aversive stimulus [51] but that may also interact with the pheromone-sensitive OR19a in the trichoid sensilla [64].

As for the response to water, in our opinion, results showing that LRRK2 mutants are trapped by water in the 1-hexanol experiments and especially the absence of difference between water and the odour in the case of 1-linalool point to an impairment in the discrimination capability of LRRK2 mutants. In fact, even if mutants present a higher electrophysiological response to both stimuli with respect to WT, the percentage of mutants which goes to the odours under a double-choice situation is about half that of WT flies. These results can be attributable to an impairment we found in the mitochondrial morphology in the antennal lobes [43], where olfactory neurons project.

### 4.3. Morphological Observations

Our observations on antennal flagellum of* Drosophila* WT males showed the presence of four types of sensilla as previously reported [65] and mapped for this species [53]; further, the LRRK^WD40^ mutant did not display noticeable gross morphological differences with respect to the WT, but a general increase in the number of sensilla in the central part of the flagellum was highlighted. As far as we know, no data are available to correlate the number of sensilla with LRRK function; we recall that LRRK2 is known to be involved in cytoskeleton assembly [22], but how a deletion of WD40 domain can be correlated to an increase of sensilla number is hard to hypothesize. In this respect, we recall that researches on* lozenge Drosophila* mutants showed changes at different extent in morphological features of antennal sensilla, including a reduction or an increase in the number and/or lack of some sensilla [66].

### 4.4. Concluding Remarks

Our functional approach to merging olfactory sensory electrophysiology with behavioural response was used to go in depth in the analysis of odour detection with the aim of considering olfaction as a diagnostic marker for PD in the LRRK^WD40^.

Our results show that, despite the dose-response relationship and the unexpected high EAG amplitude which point to a “normal” olfactory function, the LRRK^WD40^ mutants present an impairment in the behavioural response to odours. In this respect in a pioneeristic work on human odour stimuli detection in correlating electrophysiological activity from human olfactory bulb and the subjective response to stimuli, Hughes et al. [67] stated “the significance of this type of correlation remains questionable as long as there is no indication of the total response of the organism.”

In conclusion, our results suggest that olfactory behavioural impairment is a common feature for two* Drosophila* PD models such as LRRK^WD40^ and PINK1^B9^ [46] despite the two opposite electrophysiological peripheral responses and highlight the fact that* Drosophila* is a powerful model also for the LRRK2* loss-of-function* variant.

## Figures and Tables

**Table 1:** Average number (±SD) of the three types of sensilla present in a sample area (1,000 *μ*m^2^) in the central part of the flagellum of *D. melanogaster *WT and LRRK^WD40^ mutants (Student's *t*-test, 95% confidence level).

Type of sensilla	Wild type (*n* = 7)	LRRK^WD40^ (*n* = 8)	*P*
Trichoid sensilla	8.86 ± 1.06	11.50 ± 1.07	0.00036
Small basiconic sensilla	2.00 ± 0	2.63 ± 0.74	0.04549
Grooved sensilla	0.86 ± 0.38	1.50 ± 0.53	0.02003

## Abbreviations

Dm: *Drosophila melanogaster*
EAG: Electroantennogram
LRRK2: Leucine-rich repeat kinase 2
LRRK^WD40^: LRRK* loss-of-function* in the WD40 domain
PD: Parkinson's disease
PINK1^B9^: PTEN-induced putative kinase 1
WT: Wild type.
